# Reference standard for the prevention and management of hospital falls: a multidisciplinary Delphi consensus study

**DOI:** 10.1136/bmjopen-2025-105950

**Published:** 2025-10-06

**Authors:** Meg E Morris, Catherine M Said, Terry Haines, Hazel Wei Fen Heng, Frances Batchelor, Alison M Hutchinson, Jonathan P McKercher, Adam Ivan Semciw, Anne-Marie Hill, Stephen Peterson, Richard Kane, Sally Fowler-Davis, Sarah Campbell, Catherine Sherrington, Julia Gilmartin-Thomas, Uyen Phan, Claire Thwaites, Hospital Falls Reference Standard Consensus Group, Morag Taylor

**Affiliations:** 1 Academic and Research Collaborative in Health, La Trobe University, Bundoora, Victoria, Australia; 2 The Victorian Rehabilitation Centre, Healthscope Limited, Glen Waverley, Victoria, Australia; 3 Faculty of Medicine, Dentistry and Health Sciences, The University of Melbourne, Melbourne, Victoria, Australia; 4 Western Health, St Albans, Victoria, Australia; 5 Rehabilitation Ageing and Independent Living (RAIL) Research Centre, Monash University, Frankston, Victoria, Australia; 6 Department of Allied Health, Northern Health, Epping, Victoria, Australia; 7 School of Allied Health, Human Services and Sport, La Trobe University, Melbourne, Victoria, Australia; 8 National Ageing Research Institute, Melbourne, Victoria, Australia; 9 Centre for Quality and Patient Safety Research, Institute for Health Transformation, Deakin University School of Nursing and Midwifery, Melbourne, Victoria, Australia; 10 School of Allied Health, The University of Western Australia, Perth, Western Australia, Australia; 11 Western Australian Centre for Health and Ageing, The University of Western Australia, Perth, Western Australia, Australia; 12 St Vincents Hospital Melbourne, Melbourne, Victoria, Australia; 13 School of Allied Health and Social Care, Anglia Ruskin University, Cambridge, UK; 14 School of Public Health, Faculty of Medicine and Health, The University of Sydney, Camperdown, New South Wales, Australia; 15 Sydney Musculoskeletal Health, Institute for Musculoskeletal Health, The University of Sydney, Sydney, New South Wales, Australia; 16 Allied Health Department, Alfred Health, Melbourne, Victoria, Australia; 17 La Trobe University, Melbourne, Victoria, Australia

**Keywords:** Hospitals, Quality Improvement, Wounds and Injuries, Health Services

## Abstract

**Abstract:**

**Background:**

Hospital falls persist as a major threat to patient safety. This study aimed to develop an interprofessional reference standard to prevent, manage and report hospital falls.

**Methods:**

A Delphi consensus methodology, informed by the Conducting and Reporting Delphi Studies guideline, was used to design the reference standard. An interprofessional expert panel (n=47) of health professionals, researchers, policymakers and consumers participated in three Delphi rounds. Following the review of clinical guidelines, an e-Delphi survey was developed and piloted to derive 60 initial items for the standard. Two iterative rounds of e-Delphi surveys were distributed via Research Electronic Data Capture and included free-text questions and 9-point Likert scales. An online consensus meeting followed, to ratify the final standard.

**Results:**

In the first Delphi round, there was over 80% agreement for 44/60 items to be included in the reference standard. This increased to 48/60 items in Round 2. At the final consensus meeting, 12 items still did not reach consensus for inclusion and one was added, yielding 49 items. Items that replicated text according to falls with injury/without injury were combined, resulting in 42 items in the final reference standard. Agreed items included: (1) brief screening of falls risk on hospital admission; (2) comprehensive falls assessment for inpatients who are older, frailer or have complex conditions; (3) single interventions (such as environmental adaptations and exercise); (4) multifactorial interventions; (5) education of patients, families and staff; (6) optimising local falls hospital policies, procedures and leadership capability; (7) optimising documentation and reporting; (8) improving accreditation processes; (9) workforce redesign to augment falls education. Items that did not reach agreement (n=12) pertained to alarms, bed rails, grip socks, artificial intelligence, volunteers and care bundles.

**Conclusion:**

This new reference standard provides a checklist for staff, patients, managers and policymakers to reduce unwanted variations in prevention, management and reporting of hospital falls.

**Trial registration number:**

ANZCTR 386960

**STRENGTHS AND LIMITATIONS OF THIS STUDY**

- This project generated a new reference standard on the prevention, management and documentation of hospital falls.
- The standard was developed by a multidisciplinary team of falls experts, hospital managers and consumers.
- The hospital falls standard provides a checklist to enable care delivery to be benchmarked against best practice.
- A limitation was the reference panel of English-speaking participants from Oceania, limiting the cultural and geographical applicability of findings.

## Introduction

 Hospital falls continue to be a persistent cause of harm, injury and distress worldwide^1^ and inflate hospital resource utilisation and associated costs.^2^,^5^ Although clinical guidelines have already been published on falls prevention in older adults,^6 7^ there is no clinical reference standard on the prevention, management and documentation of hospital falls. Reference standards can be used to calibrate local clinical practice against agreed ‘gold-standard’ benchmarks for service delivery, policies and procedures.^8 9^ They succinctly state expectations for the diagnosis, evaluation, monitoring, comparison and documentation of healthcare delivery to optimise quality and safety.^9^

Reference standards differ from clinical guidelines because they state the level and type of care delivery that patients can expect from hospitals, rather than reviewing, collating and interpreting the research literature on known levels of evidence for different therapeutic interventions. Hospital reference standards are short, accessible, evidence-informed statements and are usually developed using an interprofessional consensus approach with health professionals, managers, consumers (service users) and research experts.^10^ They are sometimes used as a checklist by hospitals to determine if there are gaps between healthcare evidence and local practice.

Falls reference standards are needed for hospitals because falls remain one of the greatest risks to patients in hospital.^11 12^ Most health professionals, managers and policymakers are committed to preventing hospital falls.^13^,^18^ However, sometimes they may be unsure about how to implement the best practices for falls prevention, education, management, reporting, documentation and analysis.^13^,^18^ Furthermore, a knowledge gap appears to exist around how patients and staff can stop and manage hospital falls in an agreed way.

The aim of this study was to develop a consensus-based falls reference standard for use in hospitals by a range of stakeholders to improve patient safety, patient outcomes and service efficiency.

## Methods

### Research design and methodology

This study was prospectively registered (ANZCTR 386960). Participants gave informed consent at the start of Delphi Round 1. The development of this new reference standard for use in hospitals used a three-step modified Delphi group process.^19^ The study complied with the ‘Conducting and Reporting Delphi Studies (CREDES)’ guideline checklist^20^ available from the Enhancing the QUAlity and Transparency Of health Research network^21^. The group consensus process took place between December 2024 and April 2025.

Our modified e-Delphi methodology was based on the theoretical framework described by Habibi *et al.*^22^ The Delphi approach facilitates generation of group consensus while minimising dominance bias from individuals^23^,^25^ or group conformity which sometimes occurs with face-to-face panel meetings.^26^ A steering group of falls experts conducted an in-depth review of the published literature on best-available hospital falls prevention guidelines and evidence.^6 27^ This informed the development of a comprehensive list of items that was then presented to an expert panel of 47 individuals over two rounds of email questionnaires. The third round was an online face-to-face consensus meeting that was conducted to allow for expert interaction and opportunities for panel members to clarify matters or adjust views.^28^

### Steering committee and reference standard development

A steering committee of 17 expert health professionals, clinical researchers and consumers with expertise or lived experience in hospital falls prevention and management was formed through professional networks linked to the Victorian Falls Prevention Alliance (VFPA).^29^ The steering committee reviewed the 2022 World Guidelines for Falls Prevention and Management for Older Adults,^6^ the 2022 global meta-analysis of interventions specific to hospital fall prevention,^27^ a review of clinical guidelines by McKercher *et al.*^7^ and the Cochrane review by Cameron *et al*^11^ to develop an initial set of 60 consensus statements for the standards. These were then reviewed and considered by the broader consensus working group.

### Expert panel selection

Addressing hospital falls prevention and management is complex, requiring consideration of evidence-based interventions and collaboration from clinicians, hospital managers as well as consumers of healthcare.^30^ With this in mind, we aimed to select a diverse, heterogenous panel of experts to enable a broad perspective. Table 1 details the predefined criteria for inviting panel participants so that we could include research experts, health professionals, hospital administrators, policymakers and consumers of healthcare. All participants were aged over 18 years, and we sought a panel of 30–50 participants. Using direct outreach across professional networks of the VFPA and steering committee members, invitations were emailed to potential participants. Those who expressed interest in the study were sent a link to the survey that included the goals and study procedures.

**Table 1:** Inclusion criteria for Delphi panel members

Domain	Inclusion criteria
Research experts in hospital falls prevention	Authors of peer-reviewed, published primary research studies involving hospital falls prevention
Health professionals or hospital management personnel with expertise in hospital falls prevention	Registered healthcare professional with at least 1 year of experience working in a hospital and involved in hospital falls prevention
Consumers who were users of hospital care	Experience as a patient or carer of a patient with a history of hospital admission within the past 5 years

From 54 invitations sent, 47 participants consented and agreed to form the Delphi panel consensus working group. The participants who responded to the initial expression of interest email were included in subsequent Delphi rounds.

### Patient and public involvement

Consumers of healthcare who had experienced hospitalisation or cared for someone in hospital within the preceding 5 years were recruited and involved in the project. Some consumers contributed via a dedicated working group that participated in the Delphi rounds and consensus meetings. Other consumers were coauthors and contributed to study design, interpretation of the findings, manuscript writing and planning dissemination. The perspectives and lived experience of the consumers helped to contextualise the relevance of the research.

### Delphi round 1

Using Research Electronic Data Capture (REDCap) electronic data survey distribution software hosted by La Trobe University,^31 32^ a participant information statement detailing the objectives of the study and instructions was sent to each panel member in December 2024. The participants were provided with the World Falls Guidelines^6^ and a hospital falls meta-analysis^27^ which graded evidence for different falls interventions. They were then invited to vote on all 60 statements on a 9-point Likert scale ranging from 1 (not at all important), to 9 (very important). Agreement was defined a priori as ≥80% consensus for individual statements in the highest tertile (score of 7–9). This decision rule was based on a prior recommendation that a minimum of 80% agreement is necessary for expert groups of more than 10 participants to achieve content validity.^33^ The statements that did not reach consensus in Round 1 were flagged for Round 2 for review and discussion.^34^ For each statement, participants were able to provide open-ended comments, which were subsequently used to assist in score interpretation and develop revisions of statements for Round 2.

### Delphi round 2

Statements not meeting 80% consensus were reworded for clarity based on feedback from respondents. They were then redistributed to participants using REDCap in March 2025, along with the group ratings and anonymised example comments from Round 1 and best-available evidence supporting or refuting the statement. The participants were asked to rate the remaining 16 items along a 9-point Likert scale, rating statements from 1 (strongly disagree) to 9 (strongly agree). This revised Likert scale was in response to consensus agreement from participants to improve usability. The same methods for response analysis in Round 1 were applied in Round 2, with items not reaching consensus retained for group discussion in the final round.

### Delphi round 3

An online, face-to-face meeting was conducted with panel members, who were encouraged to reflect on and discuss the remaining statements for which there was no consensus. Voting in this round was through a show of electronic hands without preservation of anonymity.^35^ One further item pertaining to medication reviews was presented at this meeting with a show of electronic hands used to vote on inclusion following group discussion.

## Results

A total of 38 of the expert respondents participated in the Delphi rounds and the other authors participated in project design, analysis and reference writing. In the first round, 25 identified as female and 13 as male. Four of the participants were consumers with lived experience of hospital admission. Many of the health professionals, managers or policymakers were affiliated with the VFPA. 34 were from Australia and 4 were international experts (2 from the USA, 1 from England and 1 from Italy). 23 (67.6%) of the health professional respondents reported more than 20 years of experience working in healthcare. Nine (26.5%) of the respondents reported between 11 and 20 years of healthcare experience and three (8.8%) had less than 10 years of experience as a health professional. Eight respondents (23.5%) currently worked in hospital management roles. The clinical professions of Delphi panel participants included nursing, midwifery, medicine, physiotherapy, occupational therapy and pharmacy.

A list of the items agreed on in Round 1 plus the percentage agreement is given in table 2. The items agreed on in Round 2 plus the percentage agreement are given in table 3. For Delphi Round 3, no additional items were agreed on for inclusion in the final reference standard. Table 4 shows the excluded items and example comments from respondents, including some of the reasons for their exclusion.

**Table 2:** Delphi Round 1 items reaching consensus, with percentage agreement

Item	Agreement
**Falls risk screening and assessment**	
At hospital admission, falls risk screening occurs and falls prevention and mitigation strategies are implemented	88%
Comprehensive falls assessment occurs during hospital admission for patients that need it (older people, complex needs, person who had had multiple falls, frail)	88%
Comprehensive assessment (if needed) occurs within 3 days of hospital admission	86%
**Interventions to prevent falls**	
**Patient education**	
Patient/carer involvement in hospital falls education	97%
Patient/carer involvement in hospital falls goal setting	97%
Patient/carer involvement in management of hospital falls	100%
Patient falls education within 48 hours of admission to hospital	91%
Patients receive falls educational resources while in hospital	88%
Falls resources are tailored to the needs of people with delirium, dementia or cognitive impairment	94%
**Staff education, training and resources**	
During orientation, new staff are educated about hospital falls prevention, management, documentation and use of falls data	94%
Hospital falls education for staff occurs at regular in-services/training sessions	100%
Hospital staff are provided with online falls education and training	82%
Hospital staff have access to educational resources for falls prevention and management	94%
Hospital managers have access to falls policies, educational resources, education and training	88%
Hospital wards have ‘falls champions’	91%
Clinical educators receive training on falls prevention, management and documentation	82%
**Single interventions**	
Fast responses to call bells occur in ward	94%
Supervision or assistance with toileting is given when needed	100%
Gait aids are within reach and used when ambulating	97%
Mobilisation, exercises or physical activity	94%
Use of appropriate footwear	91%
Use glasses if required	94%
Use hearing aid if required	83%
Environmental adaptations are made if needed, such as reducing clutter, ensuring safe bathrooms, toilets, floors, pathways, lighting, furniture, rails	94%
Evidence-based management occurs for delirium, dementia and cognition	91%
Do not use physical restraints as a falls prevention strategy	80%
**Multifactorial interventions**	
Where indicated by individual assessment, multifactorial interventions are implemented	88%
**Falls management following fall without injury**	
Please consider items above, plus	
Paper-based documentation of fall details following fall without injury	91%
Electronic medical record documentation of fall following fall without injury	100%
Post fall huddle within 24 hours following fall without injury	83%
Clinical staff review individual patient risk and fall prevention strategies following fall without injury	86%
Communication with next of kin following occurs fall without injury	94%
Organisational feedback to stakeholders falls and injury rates following falls without injury	94%
Evidence-based modifications to hospital policies and procedures following a fall without injury	94%
**Falls management following a fall with injury**	
Please consider items above, plus	
Paper-based documentation following a fall with injury	91%
Electronic medical record documentation following a fall with injury	97%
Post-fall huddle within 24 hours following a fall with injury	82%
Clinical staff review individual patient risk and fall prevention strategies following fall with injury	94%
Communication with next of kin occurs following fall with injury	97%
Organisational feedback to stakeholders on falls and injury rates following falls with injury	91%
Evidence-based modifications to hospital policies and procedures following fall with injury	94%
**Use of policies and guidelines**	
Use most recent clinical guidelines on hospital falls prevention and management to inform clinical decision-making and care	97%
Use nationwide standards on hospital falls prevention and management to inform clinical decision-making and care	97%
Use local and nationwide policies on hospital falls prevention and management to inform clinical decision-making and care	100%

**Table 3:** Additional items reaching consensus with percentage agreement for each Delphi round

Item	Round 1	Round 2	Round 3
Supervised allied health assistants are involved in the delivery of hospital falls education	71%	80%	80%
Access to in-person falls prevention training for health professionals	74%	92%	92%
Quick screening of falls risk by health professionals on admission without assigning score	66%	80%	80%
External auditing or accreditation used to monitor falls rates, risks, policies and mitigation interventions	71%	96%	96%
*Medication review and screen all patients for falls risk-increasing drugs and deprescribe as appropriate	–	–	96%

* Item added at Round 3 Delphi consensus meeting.

**Table 4:** Items not reaching consensus for inclusion in the standard, with example comments

Item	Agreed with statement at Round 1	Agreed with statement at Round 2	Respondent comments (examples from range of views)
Clinicians have access to a falls community of practice	68%	64%	‘… it doesn’t seem feasible… we would need CoPs for multiple things such as pressure care, functional ability, medication…’ ‘Helpful to share resources and benchmark to identify opportunities for improvement.’
Website available for clinicians with falls resources	65%	68%	‘… resources, especially online, are difficult for clinicians working on the floor to access.’ ‘… important to have the website and resources available, for if and when they want to access.’
Hospital volunteers are trained and supervised to deliver education	45%	24%	‘… using staff such as health assistants makes more sense.’ ‘We use trained volunteers to deliver falls education, it works, no issues.’
Use low beds to prevent falls	54%	28%	‘… more about limiting injury from rolls out of bed. If trying to stop people leaving bed, then this is a restrictive care practice.’ ‘… there is still a place for low-low beds in certain circumstances…’
Use bed rails to prevent falls	54%	8%	‘…Not realistic or safe to never use but dangerous to use routinely. Needs to be individualised.’ ‘Guidelines recommend against use as a falls prevention strategy and is deemed a restrictive intervention.’
Use of grip socks	31%	8%	‘Grip socks offer false security to both patients and staff.’ ‘Grip socks increase infections and don’t reduce falls.’
Use chair alarms to prevent falls	23%	8%	‘Often, they are not turned on and response times are often too long to prevent a fall.’ ‘Unless there is a clinician nearby when the alarm goes off, it is not a falls prevention strategy.’
Use bed alarms to prevent falls	23%	12%	‘They do alert the nurse that the patient is moving. This may or may not prevent the fall. They are not a standalone intervention.’ ‘They should not be used as overarching fall prevention strategy but may have value in individual, tailored approaches.’
Use webcams to prevent falls	26%	8%	‘…many falls occur in bathroom and surveillance would not be appropriate.’ ‘Does not help you still have to get to the patient - intentional rounding and good toileting plans are more effective.’
Multifactorial fall prevention ‘Care bundles’ delivered to all patients	54%	64%	‘Care bundles should be basic, standard care practice.’ ‘There is also potential overlap with targeted, multifactorial intervention program approaches which may only be of marginal benefit if at all.’
Multifactorial fall prevention ‘Care bundles’ delivered only in high-risk settings	27%	4%	‘… many wards have overflow patients and anyone who is post-operative or on different medication to usual is at risk of falls.’ ‘Anyone, no matter their age or physical condition, good or bad, can be at heightened risk of a fall in hospital.’
Use of artificial intelligence to analyse and report hospital falls data	38%	28%	‘Don’t be scared of AI, just because we don’t know what it can and can’t do. Plan for the future.’ ‘AI has great potential but needs further testing for this setting and purpose. Agree that predicting who falls and risk factors does not translate into prevention strategies.’

In the first Delphi round, there was over 80% agreement for 44 items to be included in the reference standard, increasing to 48 items in Round 2, then 49 items after the consensus meeting (tables2  3). At the final Delphi consensus meeting, 12 of the original items still did not reach consensus for inclusion. It was agreed by the steering committee plus the attendees at the consensus meeting that these 12 statements should not be included in the final reference standard (table 4).

Final agreement for items in the reference standard included: (1) fast, brief screening of falls risk within 48 hours of hospital admission without assigning a falls risk score; (2) comprehensive falls assessment for older or frail inpatients or those with multimorbidity; (3) some single interventions to prevent and manage falls; (4) multifactorial falls prevention and management while in hospital; (5) education of patients, families and health professionals on how to prevent and manage hospital falls; (6) improving local hospital policies, procedures and leadership capability to prevent falls and injuries; (7) optimising documentation, reporting, analysis and actions arising from hospital falls data; (8) revising accreditation processes to reflect evidence-based research; (9) workforce redesign supporting trained assistants to augment falls. Our reference standard also included an a priori statement on medication-related falls screening as this was detailed previously in an evidence-based meta-analysis.^36^

A copy of the reference standard (after minor editing by the authors to enhance readability) is published in (online supplemental appendix). For the final step in deriving the standard, the items that replicated text according to falls with or without injury were combined into a single statement to reduce replication. This reduced the number of items to 42.

## Discussion

Reference standards on hospital falls can help to provide a benchmark to direct staffing and resources towards agreed methods of falls prevention and management to improve the quality of hospital service delivery. This is important given that falls are a major source of preventable adverse events in hospitals across the world^12 37^ and very costly.^2^

Our Delphi study showed that stakeholders believe that much can be done by hospital regulators, hospital leaders, interprofessional teams, individual staff and consumers who are end-users of services to tackle the persistent and debilitating problem of hospital falls and fall-related injuries. For example, in agreement with McLennan and colleagues,^38^ there was strong support for empowering hospital patients and families to be involved in falls education, goal setting and implementing targeted actions after the first fall to prevent future falls (table 2). This also agreed with the findings of randomised hospital trials by Hill *et al*^15^ and Haines *et al*.^39^ Also, fast screening for falls risk on admission without assigning a risk score^40 41^ was supported by the consensus panel, as was a detailed falls assessment for hospital patients who are very frail, old or with complex needs.^42^ Also supported were environmental modifications to prevent falls such as reducing clutter and ensuring safe bathrooms, toilets, floors, pathways, lighting, furniture and rails.^43 44^ Other reference items that corroborated with prior published research included exercises and physical activities to keep people moving^45^; safe footwear^46^; safe use of assistive devices such as wheeled walkers^27 47^; multifactorial interventions^6^; post-fall huddles soon after a fall^42 48^; medication reviews as part of multifactorial care^36 49^ and optimisation of local policies and accreditation procedures.^7^ Multifactorial falls prevention ‘bundles’ where the same cluster of interventions is given uniformly to all hospital patients did not reach consensus agreement, even though they might be useful to trial in some hospitals.^50^ The lack of consensus was in line with the findings of the large ‘6-PACK’ cluster randomised trial by Barker *et al*^51^ which showed standardised falls bundles not to be superior to usual care.^51^ In contrast, individually tailored multifactorial falls prevention interventions stacked and tailored to mitigate individual risk factors had 88% agreement from our panel, in line with the Cochrane review by Cameron *et al*.^11^

Several other items in our Delphi process did not reach consensus. For example, although artificial intelligence (AI) can evaluate data from electronic medical records to evaluate falls risk^52^ and make recommendations for risk mitigation strategies based on machine learning applied to existing datasets,^53^ our panellists did not uniformly agree that it should be mandated in hospital falls reference standards. AI is a comparatively new intervention for hospital falls prevention and might not currently be on the radar of some stakeholders. Another example of items that did not reach 80% consensus was bed and chair alarms to prevent hospital falls. A minority of panel experts agreed that use of bed and chair alarm sensors prevented hospital falls even though sensors can indicate when a patient has fallen (table 4). A large, randomised trial by Shorr *et al* reported that increased use of bed alarms in hospitals had no significant effect on fall-related events.^54^ Of interest, the item on divesting from bedside rails did not reach consensus even though rails can be considered to be a form of physical restraint. In some countries, there are laws against using physical restraints in hospitals, unless under exceptional circumstances.^55^ Of note is that all the items failing to reach consensus are practical solutions that may be considered for use with a selected care plan but cannot be considered as a standardised practice. These areas may benefit from targeted research or pilot testing before inclusion in future standards.

There were several limitations of the current study. We set the reference standard statement agreement rates at 80% whereas some recent Delphi studies set consensus as 75% agreement^49^ or lower.^56 57^ This meant that we did not include a recommendation against the use of bedrails as a falls prevention method, despite the world clinical guidelines on falls prevention for older adults advising this should be done.^6^ It may be necessary for guidelines to inform practitioners about unsafe practices that are in common use so that de-implementation can be undertaken as a service level improvement. Also, the panel did not examine the effects of poor sleep on hospital falls, even though it is known that noise, pain and medical conditions can affect sleep duration, sleep efficiency and sleep quality and therefore may contribute to related falls from patient fatigue.^58^ Bed moves were not reviewed by our panel and it is noted that Toye *et al*^59^ showed the risk of hospital falls to be correlated with the number of times a patient’s bed is moved from one room to another.^59^ The panel was composed exclusively of English-speaking participants from high-income countries, with most from Australia, potentially limiting the cultural and geographical applicability of findings. Whether or not the recommendations can be generalised to other countries or cultures awaits confirmation. Finally, it was beyond the scope of the current project to decide which member of the multidisciplinary team should lead the falls screening, assessments, interventions and documentation. It is recommended that this be a local decision given that staffing and roles vary widely according to the type of hospital and the country where hospital services are delivered.

## Conclusion

Hospital falls and associated injuries continue to be a global challenge for healthcare systems. This reference standard for hospital falls was generated by multiple stakeholders, to provide a practical resource that aims to reduce unwanted variations in how hospital falls are prevented and managed. Future work should explore the reference standard implementation and impact on hospital falls and patient outcomes.

## Supplementary material

10.1136/bmjopen-2025-105950 online supplemental file 1

## Data Availability

Data are available upon reasonable request to the lead author.
