# Supplementary material for: Reference standard for the prevention and management of hospital falls: a multidisciplinary Delphi consensus study
Source: BMJ Open. 2025 Oct 6;15(10):e105950. doi: 10.1136/bmjopen-2025-105950 (PMC12506041; doi:10.1136/bmjopen-2025-105950)
Supplement: online supplemental file 1 [file bmjopen-15-10-s001.pdf]

Reference standard for the prevention and management of hospital falls

		TICK OR CROSS	COMMENTS
**(a) Falls Risk Screening and Assessment**			
**1.**	At hospital admission, falls risk screening occurs, and corresponding falls prevention and mitigation strategies are implemented	☐	
**2.**	Quick screening of falls risk is completed by health professionals on admission without assigning a risk score	☐	
**3.**	Comprehensive falls assessment occurs during hospital admission for patients that need it such as older people, those with complex needs, multi-morbidity, frailty, frequent fallers	☐	
**4.**	Comprehensive assessment (if needed) occurs within 3 days of admission	☐	
**5.**	All patients receive a medication review and are screened for drugs that increase falls risk; medications deprescribed, as appropriate	☐	
**(b) Interventions to Prevent Falls**			
**Patient and Carer Education**			
**6.**	Patient and carers (as appropriate) are involved in hospital falls education	☐	
**7.**	Patient and carers (as appropriate) are involved in falls goal setting	☐	
**8.**	Patient and carers (as appropriate) are involved in the management and prevention of hospital falls	☐	
**9.**	Patient falls education is provided within 48 hours of hospital admission	☐	
**10.**	Patients receive falls educational resources whilst in hospital	☐	
**11.**	Falls resources are made available that are tailored to the needs of people with delirium, dementia, cognitive impairment	☐	
**12.**	Trained and supervised allied health or nursing assistants are involved in the delivery of hospital falls prevention education for patients	☐	
**Staff Education, Training and Resources**			
**13.**	During staff onboarding and orientation, new staff are educated about hospital falls prevention, management, documentation and falls data	☐	
**14.**	Hospital staff falls education occurs at regular in-services and training	☐	
**15.**	Hospital staff are provided with online falls prevention and management, education and training	☐	
**16.**	Hospital staff have in-person falls prevention and management training	☐	
**17.**	Staff have access to educational resources for falls prevention and management	☐	
**18.**	Hospital managers have access to up-to-date hospital falls policies, educational resources, education and training	☐	
**19.**	Hospital wards have "falls champions"	☐	
**20.**	Clinical educators receive education and training on hospital falls prevention, management and documentation	☐	

	TICK OR CROSS	COMMENTS
**Single Interventions**		
**21.** Fast responses to call bells occurs on wards	☐	
**22.** Supervision or assistance with toileting is given when needed	☐	
**23.** Gait assistive devices (such as walking frames and sticks) are within reach and used when ambulating	☐	
**24.** Patient mobilisation, exercises or physical activities occur in hospital (as appropriate)	☐	
**25.** Patients use appropriate footwear	☐	
**26.** Patients use glasses if required	☐	
**27.** Patients use hearing aids if required	☐	
**28.** Environmental adaptations are made, such as reducing room clutter, and ensuring safe bathrooms, toilets, floors, pathways, lighting, furniture, rails, ramps etc.	☐	
**29.** Evidence-based management occurs for delirium, dementia and cognitive impairment	☐	
**30.** Physical restraints are not used as a fall prevention intervention	☐	
**Multifactorial Interventions**		
**31.** Where indicated by individual assessment, multifactorial interventions are implemented	☐	

(c) Falls Management Following a Fall With or Without Injury

**32.** Electronic medical record documentation of fall details and any injuries is completed (if the hospital has an electronic medical record)	☐	
**33.** Paper based documentation of fall details, and any injuries, is completed (if required by the hospital)	☐	
**34.** Post fall huddle occurs within 24 hours following a fall	☐	
**35.** Clinical staff complete a review of individual patient falls risk and fall prevention strategies following a fall	☐	
**36.** Communication with next of kin occurs following a fall	☐	
**37.** Organisation provides feedback to staff and other stakeholders detailing falls rates and injury rates	☐	
**38.** Evidence-based modifications are made to hospital falls policies and procedures when required	☐	

(d) Falls Policies and Guidelines

**39.** The most recent clinical guidelines on hospital falls prevention and management are used to inform clinical decision making and care	☐	
**40.** Nation-wide standards on hospital falls prevention and management are used to inform clinical decision making and care	☐	
**41.** Local and nation-wide policies on hospital falls prevention and management are used to inform clinical decision making and care	☐	
**42.** External auditing or accreditation are used to monitor falls rates, risks, policies, interventions, and documentation.	☐	
