# Supplementary material for: Reference standard for the prevention and management of hospital falls: a multidisciplinary Delphi consensus study
Source: BMJ Open. 2025 Oct 6;15(10):e105950. doi: 10.1136/bmjopen-2025-105950 (PMC12506041; doi:10.1136/bmjopen-2025-105950)
Supplement: Supplementary file 2 [file bmjopen-2025-105950.reviewer_comments.pdf]

PEER REVIEW HISTORY

BMJ Open publishes all reviews undertaken for accepted manuscripts. Reviewers are asked to complete a checklist review form and are provided with free text boxes to elaborate on their assessment. These free text comments are reproduced below.

ARTICLE DETAILS

Title (Provisional)

Reference Standard for the Prevention and Management of Hospital Falls: A Multidisciplinary Delphi Consensus Study

Authors

Morris, Meg; Said, Catherine M; Haines, Terry; Heng, Hazel Wei Fen; Batchelor, Frances; Hutchinson, Alison M.; McKercher, Jonathan P.; Semciw, Adam Ivan; Hill, Anne-Marie; Peterson, Stephen; Kane, Richard; Fowler-Davis, Sally; Campbell, Sarah; Sherrington, Catherine; Gilmartin-Thomas, Julia; Phan, U; Thwaites, Claire; Hospital Falls Reference Standard Consensus Group, On behalf of

---

VERSION 1 - REVIEW

**Reviewer**	**1**
**Name**	**Arnold, Susanne**
**Affiliation**	**Warwick Medical School, Warwick Clinical Trials Unit**
**Date**	**18-Jul-2025**
**COI**	**None**

This is a review of the Reference Standard for the Prevention and Management of Hospital Falls: A Multidisciplinary Delphi Consensus Study.

Abstract: A clear summary.

Introduction: A very clear overview of the project and the need for a reference standard.

Methods:

Page 9 - line 171: Can you give the full title of CREDES before the abbreviation.

Page 9 - line 175: Is there a word missing? Should it say Our modified e-Delphi methodology WAS based on the theoretical framework described by Habbibi et al. And you have misspelt 'whilst' at the end of that line.

Page 10 - line 193: here you describe and initial set of 61 consensus statements but on page 11 you state that participants were invited to vote on all 60 statements - which is correct - from all your subsequent numbers I think it is 60?

Page 11 - lines 228-229: the participants were asked to rate the remaining 16 statements... feels like it should be in the results not the methods.

Page 12 - lines 234 -240: this section on Delphi round 3 also feels like you are describing some results and some methods?

Results:

Page 13 - line 260: repetition of the word 'in'

Table 2 is really helpful and so interesting. Just out of interest, was there any discussion as to who i.e. which member of the MDT would be leading on these assessments, interventions and documentation?

Discussion:

I think my previous point might need addressing in the discussion. Although it feels like it should be a MDT approach (and rightly so), if staff are using this as a benchmarking there may need some more specifics about who you would be expecting to complete some of these standards or do you think this would need to be worked out locally and therefore cannot be part of the reference standard?

If you have the word count do you think you could tell the readers about the plans for this reference standard i.e. who you intend to share it with and where do you plan to implement it (if that has been decided).

**Reviewer**	**2**
**Name**	**Roberti, Javier**
**Affiliation**	**Universidad Nacional de Entre Rios**
**Date**	**01-Aug-2025**
**COI**	**None**

This manuscript addresses an important topic in falls prevention by using a structured Delphi consensus process to develop expert recommendations. The study is well reported and I have some suggestions and questions to improve its readability and clarity..

Page 10, line 187, description of steering committee of 14 expert health professionals, please describe the inclusion criteria to be considered experts.

In the same section, add some more details on the process used to select the 61 statements, because the steering committee was quite large.

Did you conduct some kind of scoping review to be able to identify those references used to select the 61 statements?

Was there any grading for the statements sent to experts in relation to the level or quality of evidence supporting each statement?

For the expert panel, Please reference to table 1 for the inclusion criteria of experts. Mention something in the text. This is critical for expert panels.

Please provide some information on the context given to experts in relation to where the standard would be applied.

In methods, please explain if patients and carers were involved and why. The journal may have asked about this point in the submission process.

In Round 1, you mention there were 60 statements instead of the previous 61. Please correct.

I would like to know a bit more about the form sent to experts. Did you include references for each statement? Any justification? Any further explanation for the likert to be able to vote?

In Round 2, why did the Likert scale changed from importance to agreement?

In Round 2, when you distributed the results of round 1, I imagine this was anonymous and the recipient could only identify their own response. Please mention this.

In Round 3, was there any explanation to eliminate the last 12 statements? How did experts vote or decide this? Was it that there was agreement, ie more than 80% voting on the lower tertile?

Please mention if participants gave any consent. The journal may ask for a subheading for ethics.

In results, you mention 38 participants, but you mention 47 had accepted the invitation with no withdrawals. Please clarify.

I dont think consumer is the best word here, perhaps user.

PAge 11, line 260 the word in is repeated.

In results you mention that at the final consensus meeting 12 items did not reach consensus for inclusion, following my previous comment, was there consensus for exclusion? Or the steering committee decided to exclude them because there was no consensus?

Was there any discussion about grading the statements after consensus according to impact or feasibility for example?

When reading the final list of recommendations, I see that some of them are broad and may need rephrasing to be more specific and, therefore, more actionable.

In Table 4, you use Percentage categories, %R1 agree, R2 agree, R2 neutral, R2disagree. This is not clear. Please explain them briefly when you describe the consensus definition for clarity and as a note in the table.

This is a valuable contribution to the field. With some clarifications, the manuscript will be strengthened and more impactful.

VERSION 1 - AUTHOR RESPONSE

**Reviewer 1 comments**	**Response**
**Abstract**: A clear summary.<br>**Introduction**: A very clear overview of the project and the need for a reference standard.	Thank you for your feedback.
**Methods:** Page 9 - line 171: Can you give the full title of CREDES before the abbreviation.	This has now been added: "Conducting and Reporting Delphi Studies (CREDES)"
Page 9 - line 175: Is there a word missing? Should it say Our modified e-Delphi methodology WAS based on the theoretical framework described by Habbibi et al. And you have misspelt 'whilst' at the end of that line.	Thank you for picking up the missing word we have now added. (was)
Page 10 - line 193: here you describe and initial set of 61 consensus statements but on page 11 you state that participants were invited to vote on all 60 statements - which is correct - from all your subsequent numbers I think it is 60?	Thank you for raising this. As detailed in methods lines 238-240 and in the abstract line105, there was an initial 60 items that went through Delphi round 1 and 2, with a final additional item (n=61) (medication review) raised for group discussion and voting via show of hands at the final Delphi round 3 consensus meeting. We can see the error and have now revised this to 60 statements.
Page 11 - lines 228-229: the participants were asked to rate the remaining 16 statements... feels like it should be in the results not the methods.	Thank you for this suggestion. We have decided to keep this sentence in the methods as it is procedural rather than data or a finding.
Page 12 - lines 234 -240: this section on Delphi round 3 also feels like you are describing some results and some methods?	Noted and we have moved the following sentence to the Results section: which now adds: "It was agreed these 12 statements should be eliminated from the final consensus reference standard (Appendix 2)".
**Results:** Page 13 line 260: repetition of the word 'in'	The extra "in" has now been removed
Table 2 is really helpful and so interesting. Just out of interest, was there any discussion as to who i.e. which member of the MDT would be leading on these assessments, interventions and documentation?	It was beyond the scope of this project to decide which member of the MDT would be leading on these assessments, interventions and documentation, especially as roles varies across hospitals. In some hospitals it is doctors, in others nurses and others allied health professionals. We have elaborated on this more in the revised Discussion (last 2 sentences).
**Discussion:** I think my previous point might need addressing in the discussion. Although it feels like it should be a MDT approach (and rightly so), if staff are using this as a benchmarking there may need some more specifics about who you would be expecting to complete some of these standards or do you think this would need to be worked out locally and therefore cannot be part of the reference standard?	Agree totally and we have elaborated on this more in the revised Discussion (last 2 sentences) which now say:<br>… "Finally, it was beyond the scope of the current project to decide which member of the multi-disciplinary team should lead falls screening, assessments, interventions and documentation. It is recommended this be a local decision given that staffing and roles vary widely according to the type of hospital and the country where services are delivered".

If you have the word count do you think you could tell the readers about the plans for this reference standard i.e. who you intend to share it with and where do you plan to implement it (if decided).	We are currently working on the implementation plan and scale up. This will be the topic of our next publication thank you.
**Reviewer 2 Comments**	**Response**
This manuscript addresses an important topic in falls prevention by using a structured Delphi consensus process to develop expert recommendations. The study is well reported and I have some suggestions and questions to improve its readability and clarity.	Thank you for your feedback.
Page 10, line 187, description of steering committee of 14 expert health professionals, please describe the inclusion criteria to be considered experts.	Table 1 shows the inclusion criteria for the inclusion criteria to be considered experts.
In the same section, add some more details on the process used to select the 61 statements, because the steering committee was quite large.	This is already in the Methods section of the manuscript, in paragraph 2.
Did you conduct some kind of scoping review to be able to identify those references used to select the 61 statements?	Yes we did a meta-analyis first and published in in Age & Ageing:<br>"Morris ME, Webster K, Jones C, Hill AM, Haines T, McPhail S, et al. Interventions to reduce falls in hospitals: a systematic review and meta-analysis. Age Ageing. 2022;51(5)".
Was there any grading for the statements sent to experts in relation to the level or quality of evidence supporting each statement?	We have now added: "The participants were provided with the World Falls Guidelines [6] and a Hospital Falls meta-analysis [27] which graded evidence for different falls interventions."
For the expert panel, please reference to table 1 for the inclusion criteria of experts. Mention something in the text. This is critical for expert panels.	Yes we have done this.
Please provide some information on the context given to experts in relation to where the standard would be applied.	We are currently working on the implementation plan and scale up. This will be the topic of our next publication thank you.
In methods, please explain if patients and carers were involved and why. The journal may have asked about this point in the submission process.	Yes this is detailed in Table 1.
In Round 1, you mention there were 60 statements instead of the previous 61. Please correct.	We have now fixed this typo.
I would like to know a bit more about the form sent to experts. Did you include references for each statement? Any justification?	The form we sent had proposed items plus links to references for each of the statements.
In Round 2, why did the Likert scale changed from importance to agreement?	We have added new text to say: "This revised Likert scale was in response to consensus agreement from participants to improve usability."
In Round 2, when you distributed the results of round 1, I imagine this was anonymous and the recipient could only identify their own response. Please mention this.	We agree and have mentioned it.<br><br>…"they were then redistributed to participants using REDCap in March 2025, along with the group ratings and anonymised example comments from Round 1 and best-available evidence supporting or refuting the statement."

In Round 3, was there any explanation to eliminate the last 12 statements? How did experts vote or decide this? Was it that there was agreement, ie more than 80% voting on the lower tertile?	We have added in the methods that “Any items not reaching 80% agreement at this stage were not included in the final standard.”
Please mention if participants gave any consent. The journal may ask for a subheading for ethics.	We have added that “Participants gave informed consent at the start of Delphi Round 1”.
I dont think consumer is the best word here, perhaps user.	We agree and have changed it to user.
I dont think consumer is the best word here, perhaps user.	We agree and have changed it to user.
Page 11, line 260 the word in is repeated.	The extra “in” has now been removed
In results you mention that at the final consensus meeting 12 items did not reach consensus for inclusion, following my previous comment. Was there consensus for exclusion? Or the steering committee decided to exclude them because there was no consensus?	Good point. We have added that it was agreed by the steering committee and other participants who attended the final consensus meeting. This has now been added.
Was there any discussion about grading the statements after consensus according to impact or feasibility for example?	It was beyond the scope of the current project to do further grading of the statements after consensus according to impact or feasibility. This is the topic of a new publication we are currently writing.
When reading the final list of recommendations, I see that some of them are broad and may need rephrasing to be more specific and, therefore, more actionable.	Thankyou for this reflection. It took many months to gain consensus on the wording of the recommendations so at the current time we shall leave as it.
In Table 4, you use Percentage categories, %R1 agree, R2 agree, R2 neutral, R2disagree. This is not clear. Please explain them briefly when you describe the consensus definition for clarity and as a note in the table.	Based on your feedback we have done a minor revision of Table 4 to make it more understandable.
This is a valuable contribution to the field. With some clarifications, the manuscript will be strengthened and more impactful.	Thank you very much for your helpful feedback.

VERSION 2 - REVIEW

Reviewer 2

Name Roberti, Javier

Affiliation Universidad Nacional de Entre Rios

Date 01-Sep-2025

COI

The authors have addressed all comments. Thank you!
